# β2‐adrenergic stimulation induces interleukin‐6 by increasing Arid5a, a stabilizer of mRNA, through cAMP/PKA/CREB pathway in cardiac fibroblasts

**DOI:** 10.1002/prp2.590

**Published:** 2020-04-17

**Authors:** Shota Tanaka, Atsuki Imaeda, Kotaro Matsumoto, Makiko Maeda, Masanori Obana, Yasushi Fujio

**Affiliations:** ^1^ Laboratory of Clinical Science and Biomedicine Graduate School of Pharmaceutical Sciences Osaka University Suita City Osaka Japan; ^2^ Project of Clinical Pharmacology and Therapeutics Graduate School of Pharmaceutical Sciences Osaka University Suita City Osaka Japan; ^3^ Integrated Frontier Research for Medical Science Division Institute for Open and Transdisciplinary Research Initiatives Osaka University Suita City Osaka Japan

**Keywords:** fibroblasts, interleukin‐6, β adrenergic receptor

## Abstract

**Background and Purpose:**

In cardiovascular diseases, cardiac fibroblasts (CFs) participate in the myocardial inflammation by producing pro‐inflammatory cytokines, worsening the prognosis. β2‐adrenergic receptor (AR) and β3AR are expressed in CFs, and β‐adrenergic stimulation promotes CFs to produce pro‐inflammatory cytokines. However, the mechanism of the expression of pro‐inflammatory cytokines in response to β‐adrenergic stimulation remains to be fully elucidated.

**Experimental Approach:**

CFs were isolated from adult wild‐type or *AT‐rich interactive domain‐containing protein 5A* (*Arid5a*) knockout mice. The expression of mRNA was measured by real‐time RT‐PCR. Interleukin (IL)‐6 protein was measured by ELISA. The activity of nuclear factor‐κB (NF‐κB) and cyclic AMP (cAMP) response element binding protein (CREB) was assessed by ELISA‐like assay or Western blotting.

**Key Results:**

The β‐adrenergic stimulation remarkably induced IL‐6 mRNA and protein through β2AR in CFs. The activation of adenylate cyclase and the enhancement of intracellular cAMP resulted in the upregulation of IL‐6 mRNA expression. The induction of IL‐6 transcript by β2AR signaling was independent of NF‐κB. Concomitant with IL‐6, the expression of Arid5a, an IL‐6 mRNA stabilizing factor, was enhanced by β2‐adrenergic stimulation and by cAMP increase. Importantly, β2AR signaling‐mediated IL‐6 induction was suppressed in *Arid5a* knockout CFs. Finally, β2AR stimulation phosphorylated CREB via PKA pathway, and the activation of CREB was essential for the induction of Arid5a and IL‐6 mRNA.

**Conclusion and Implications:**

β2‐adrenergic stimulation post‐transcriptionally upregulates the expression of IL‐6 by the induction of Arid5a through cAMP/PKA/CREB pathway in adult CFs. β2AR/Arid5a/IL‐6 axis could be a therapeutic target against cardiac inflammation.

## 1 INTRODUCTION

Heart disease is one of the leading causes of death in the world, and chronic heart failure is a terminal state of almost all the cardiac diseases, such as myocardial infarction, valvular diseases, and myocarditis. It is well known that cardiac damage triggers inflammation, which further impairs cardiac function.^1^ Among various kinds of pro‐inflammatory cytokines, interleukin (IL)‐6 has been reported to be involved in cardiac hypertrophy and fibrosis.^2^, ^3^ Interestingly, the injection of neutralizing antibody for IL‐6 ameliorates cardiac disorder induced by myocardial infarction in mice.^4^

Cardiac cells are classified into three populations: cardiomyocytes, cardiac fibroblasts (CFs), and vascular cells. CFs occupy 10%‐15% of total cardiac cells.^5^ So far, CFs have been considered to contribute mainly to the maintenance of the cardiac structure by producing extracellular matrix. However, recent studies have demonstrated that CFs regulate the cardiac function through the secretion of various cytokines and growth factors.^6^ For example, in response to angiotensin II, CFs released IL‐6 family proteins and enlarged cardiomyocytes.^7^ Thus, the suppression of the release of pro‐inflammatory cytokines from CFs could be one of the promising strategies against cardiac remodeling.

It is widely accepted that β‐adrenergic receptor (βAR) agonist promotes the production of pro‐inflammatory cytokines^8^, ^9^ and leads to the onset and/or progression of heart failure. Although adult murine CFs express β2AR and β3AR,^6^, ^10^ the signaling pathway downstream of ARs remains to be fully elucidated in CFs. Here, since biological functions of CFs depend on their maturity, we have addressed whether and how βAR stimulation induces the production of pro‐inflammatory cytokines by using adult murine CFs, not neonatal CFs that have been usually used in the previous studies.^11^, ^12^

In addition to the transcriptional regulation of mRNA expression by the transcription factors, such as nuclear factor‐κB (NF‐κB), mRNA stabilization is critical for the expression of pro‐inflammatory cytokines.^13^ For example, tumor necrosis factor (TNF)‐α mRNA is destabilized by the RNA binding protein, tristetraprolin.^14^ Interestingly, regnase‐1 and roquin promote to degrade mRNA of inflammatory factors, such as IL‐6, and double mutation mice of these two genes evoke cardiac inflammation and fibrosis.^15^, ^16^ In contrast to mRNA degradation, AT‐rich interactive domain‐containing protein 5A (Arid5a) was identified as an IL‐6 mRNA stabilizing factor.^17^ The pathophysiological significances of mRNA stability in cytokine production have been investigated mainly in immune cells; however, it is still unknown whether mRNA stabilization is involved in cytokine production in CFs.

In this study, we demonstrated that β2‐adrenergic stimulation remarkably increases IL‐6 in adult murine CFs. Moreover, Arid5a is induced by β2AR stimulation via cyclic AMP (cAMP)/PKA/CREB pathway and plays a crucial role in IL‐6 upregulation. This finding is the first demonstration that Arid5a regulates the cytokine production downstream of adrenergic signaling.

## 2 MATERIALS AND METHODS

### 2.1 Animals

The care of animals was performed in accordance to Osaka University animal care guidelines, and all animal experiments were approved by the Experimental Animal Care and Use Committee of the Graduate School of Pharmaceutical Sciences, Osaka University (approved as Douyaku 28‐14). All animal experiments were performed according to the Guide for the Care and Use of Laboratory Animals, Eighth Edition, updated by the US National Research Council Committee in 2011.

C57BL/6J (wild‐type, WT) mice were purchased from Shimizu Laboratory Supplies (Kyoto, Japan). *Arid5a* KO mice were gifted by Professor Tadamitsu Kishimoto, Osaka University.^17^

### 2.2 Reagents

Reagents used in this study included isoproterenol (Sigma Aldrich), salbutamol, forskolin, bucladesine, (Wako), CL‐316243, H89, ICI‐118551, L‐755507 (Cayman Chemical), CGP20712A (R&D Systems), and recombinant human IL‐1β (Peprotech).

### 2.3 Preparation of cardiac fibroblasts from adult mice

Cardiac fibroblasts were prepared by reference to the protocol described previously.^18^ Briefly, cardiac fibroblasts were isolated from 6‐ to 8‐week‐old male or female mice. After injection of heparin sodium (50 units/mouse; Wako), mice were anesthetized with isoflurane (Pfizer) and sacrificed to extract their hearts. The hearts were minced and digested with the buffer including collagenase B (0.025 units/mL; Roche), collagenase D (0.025 units/mL; Roche), and protease XIV (0.02 mg/mL; Sigma Aldrich) for 45 minutes. After filtration with 70 μm mesh, cell suspension was centrifuged at 300 × ***g*** for 5 minutes, followed by the resuspension with DMEM (Sigma Aldrich) with 10% FBS (Thermo Fisher Scientific) and 1% penicillin/ Streptomycin (Nakarai Tesque) and seeded in the 6 cm dish coated with laminin (Thermo Fisher Scientific). To remove cardiomyocytes, the medium was changed 1.5 hours after seeding. Cardiac fibroblasts were cultured in the incubator (37℃, 5% CO_2_/ 95% air). All CFs were used for experiments after second passage. Cells were treated with reagents 24 hours after serum depletion.

### 2.4 Real‐time RT‐PCR

Total RNA was extracted from cardiac fibroblasts with QIAzol (QIAGEN) and refined by ethanol precipitation. cDNA was synthesized from 1 μg total RNA with oligo dT (Thermo Fisher Scientific) and ReverTra Ace (Toyobo). Quantitative RT‐PCR was performed according to the manufacturer's protocol. Briefly, the amount of cDNA was measured by Applied Biosystems StepOne Real‐Time PCR system (Applied Biosystems) using Fast SYBR Green Master Mix (Thermo Fisher Scientific). The primer sequences to use quantitative RT‐PCR were as follows;

Mouse IL‐6_forward, 5′‐AAGAGACTTCCATCCAGTTGCCTTC‐3′.

Mouse IL‐6_reverse, 5′‐ATTATATCCAGTTTGGTAGCATCCATC‐3′.

Mouse IL‐1β_forward, 5′‐GACAAAATACCTGTGGCCTTGGGCC‐3′.

Mouse IL‐1β_reverse, 5′‐GAGGTGCTGATGTACCAGTTGGGGA‐3′.

Mouse TNF‐α_forward, 5′‐CCATTCCTGAGTTCTGCAAAGG‐3′.

Mouse TNF‐α_reverse, 5′‐AGGTAGGAAGGCCTGAGATCTTATC‐3′.

Mouse Arid5a_forward, 5′‐CCAAGCCCAGGAAGCAATACA‐3′.

Mouse Arid5a_reverse, 5′‐GTGGTGGAGAGGGTCCAGATA‐3′.

Mouse GAPDH_forward, 5′‐CATCACCATCTTCCAGGAGCG‐3′.

Mouse GAPDH_reverse, 5′‐GAGGGGCCATCCACAGTCTTC‐3′.

### 2.5 ELISA

IL‐6 protein level, secreted from CFs, was assessed by Mouse IL‐6 Quantikine ELISA Kit (R&D Systems) according to the manufacturer's protocol. Briefly, cell culture supernatants were applied to the assay plate coated by anti‐mouse IL‐6 antibody and incubated for 2 hours at room temperature. After washing, HRP conjugated anti‐mouse IL‐6 antibody was added to each well and incubated for 2 hours at room temperature. After developing reaction for 30 minutes, the light absorbance (450 nm) was detected with EMax Plus (Molecular Devices).

### 2.6 Western blotting analysis

Protein samples were extracted from cardiac fibroblasts with the mixture of RIPA buffer (50 mmol/L Tris‐HCl pH 7.4, 150 mmol/L NaCl, 1% NP‐40, 0.5% sodium deoxycholate, 0.1% SDS, 1 mmol/L EDTA, 1 mmol/L NaF, and 1 mmol/L Na_3_OV_4_) and 5 × SDS sample buffer (50 mmol/L Tris‐HCl pH6.8, 2% SDS, and 10% glycerol) at a ratio of 4:1 with 1% protein kinase inhibitor cocktail (Nakarai Tesque). After the measurements of the protein concentration using BCA Protein Assay Reagent kit (Thermo Fisher Scientific), 2‐mercaptoethanol was added to the protein samples at 1% final concentration. Heating samples at 95℃ for 5 minutes, SDS‐PAGE was performed for 75 minutes at 135 V using 12.5% polyacrylamide‐SDS gel. Proteins were transferred from gels to PVDF membrane Immobilon‐P (Merck Millipore) by wet‐blotting method for 90 minutes at 270 mA. The membranes were blocked with 5% BSA in TBS‐0.05% Tween20 for 1 hour at room temperature. After incubated with primary antibodies overnight at 4℃, the membranes were reacted with proper secondary antibodies conjugated with horseradish peroxidase (HRP) for 2 hours at room temperature. After developing target protein with ECL reagent (Promega), the light emission was detected with ImageQuant LAS 4010 using ImageQuant TL software (GE Healthcare). The quantification of the protein bands was performed by ImageJ software (National Institute of Health). The antibodies used in this study were as follows: Mouse anti‐GAPDH (1:4000, Merck Millipore, Cat# MAB374, RRID: AB_2107445), mouse antiphospho‐IκBα (S32/36, 5A5, 1:1000, Cell Signalling Technolog, Cat# 9246S, RRID: AB_2267145), and rabbit antiphospho‐CREB (S133, 87G3, 1:1000, Cell Signalling Technology, Cat# 9198S, RRID: AB_2561044) as primary antibodies and goat anti‐mouse IgG (1:4000, Jackson ImmunoResearch, Cat# 115‐035‐062, RRID: AB_2338504), and goat anti‐rabbit IgG (1:1000, Cell Signalling Technology, Cat# 7074S, RRID: AB_2099233) as secondary antibodies.

### 2.7 NF‐κB p65 transcription factor assay

NF‐κB activity was measured by NF‐κB p65 Transcription Factor Assay Kit (Abcam) according to the manufacturer's protocol. Briefly, nuclear extracts were prepared from cardiac fibroblasts and applied to the assay plate coated by dsDNA with p65 binding region at 4℃ overnight. After washing, the plate was incubated with primary antibody (rabbit anti‐p65, 1:100; attachment of the kit) for 2 hours at room temperature, followed by the incubation with HRP conjugated secondary antibody (goat anti‐rabbit IgG, 1:100; attachment of the kit) for 2 hours at room temperature. After developing reaction for 20 minutes, the light absorbance (450 nm) was detected with EMax Plus.

### 2.8 Statistical analysis

All statistical analyses were performed by Statcel Ver.4 (The Publisher OMS). The statistical difference between groups was determined by Student's *t* test, Dunnett test, Steel test, or Tukey‐Kramer test. Post hoc tests were performed only if the *F* values analyzed by one‐way ANOVA test were significant. *P* < .05 was considered as significant difference.

## 3 RESULTS

### 3.1 β‐adrenergic stimulation induces IL‐6 expression through β2‐adrenergic receptor

**Figure 1:**
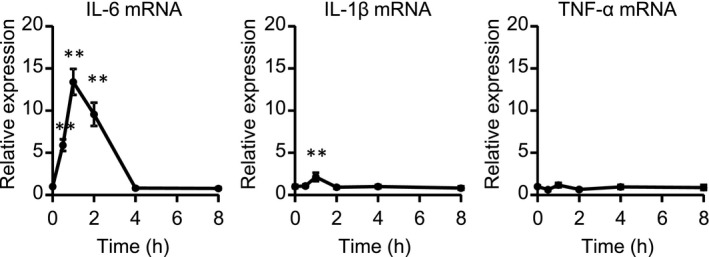
β‐adrenergic stimulation remarkably increased IL‐6 mRNA in cardiac fibroblasts (CFs) isolated from adult mice. CFs were treated with ISO (10 μmol/L) for indicated times. The expression of IL‐6, IL‐1β, and TNF‐α mRNA was measured by real‐time RT‐PCR. Results are shown as mean ± SEM (n = 6). **P < .01 vs 0 h by Dunnett test

**Figure 2:**
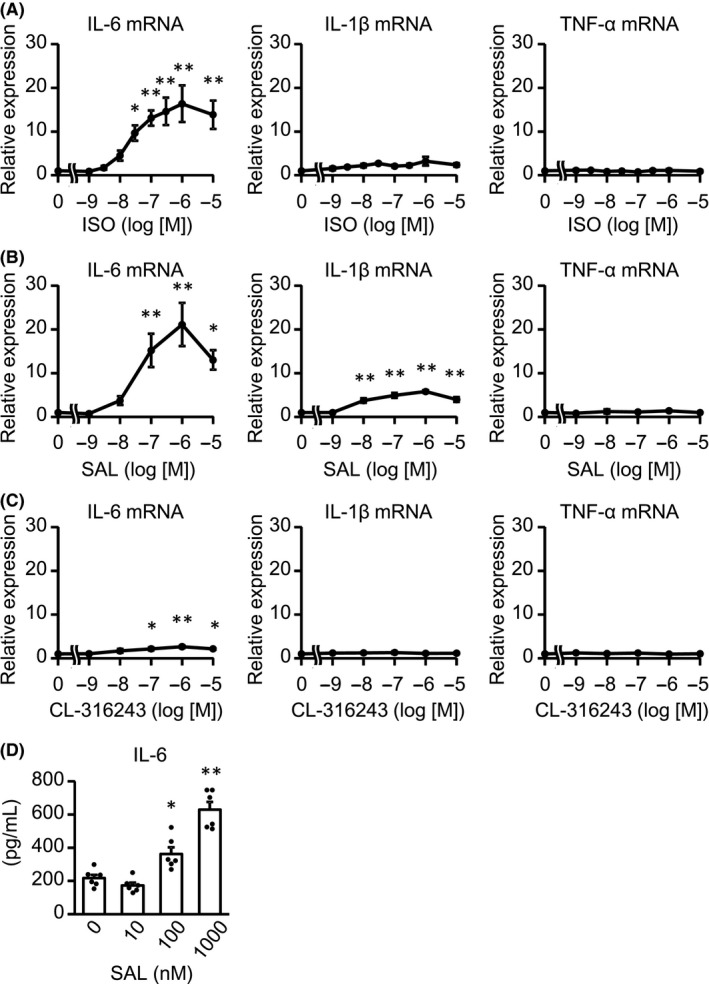
IL‐6 expression was induced predominantly by the stimulation of β2AR, not β3AR. A ‐ C) CFs were treated with the indicated concentrations of ISO (A), SAL (B), or CL‐316243 (C) for 1 h. The expression of IL‐6, IL‐1β, and TNF‐α mRNA was measured by real‐time RT‐PCR. Results are shown as mean ± SEM (A, n = 8, B, C, n = 6). *P < .05, **P < .01 vs vehicle by Dunnett test. D, CFs were treated with the indicated concentrations of SAL for 24 h. IL‐6 protein, secreted by CFs in culture medium, was measured by ELISA. Results are shown as mean ± SEM (n = 6). *P < .05, **P < .01 vs vehicle by Dunnett test

To address the mRNA expression of pro‐inflammatory cytokines, including IL‐6, IL‐1β, and TNF‐α, in response to β‐adrenergic stimulation in CFs, we stimulated CFs with isoproterenol (ISO), a nonselective βAR agonist (Figure 1). The expression of IL‐6 mRNA increased by more than 10‐fold 1 hour after ISO stimulation and decreased to baseline thereafter, while IL‐1β mRNA level was enhanced at most twice compared with baseline. In contrast, TNF‐α mRNA did not change after ISO treatment. These results suggested that βAR signaling predominantly upregulated IL‐6. Adult murine CFs mainly expressed β2AR and β3AR, but not β1AR.^10^ Thus, we investigated which subtype of βAR was responsible for IL‐6 upregulation in response to ISO (Figure 2A‐C). ISO and salbutamol (SAL), a selective β2AR agonist, increased IL‐6 mRNA in a concentration‐dependent manner (Figure 2A,B), while CL‐316243, a selective β3AR agonist, only doubled IL‐6 mRNA compared with control (Figure 2C). Of note, maximal induction of IL‐6 mRNA by SAL was similar to that by ISO, indicating that ISO‐induced upregulation of IL‐6 is mainly mediated through β2AR. As shown in Figure 1, ISO slightly increased IL‐1β mRNA, although there was no significant difference. SAL, but not CL‐316243, also increased IL‐1β mRNA with statistical significance, but to a lesser extent than IL‐6 mRNA. Expectedly, neither SAL nor CL‐316243 influenced the expression of TNF‐α mRNA. In addition, ICI‐118551, a selective β2AR antagonist, completely suppressed SAL‐induced IL‐6 mRNA upregulation. On the other hand, neither CGP20712A, a selective β1AR antagonist, nor L‐755507, a selective β3AR antagonist, affected IL‐6 mRNA level (Figure S1). Finally, ELISA analysis showed that β2‐adrenergic stimulation with SAL increased IL‐6 protein secreted by CFs in a concentration‐dependent manner (Figure 2D).

### 3.2 The increase in camp also enhances IL‐6 mRNA

**Figure 3:**
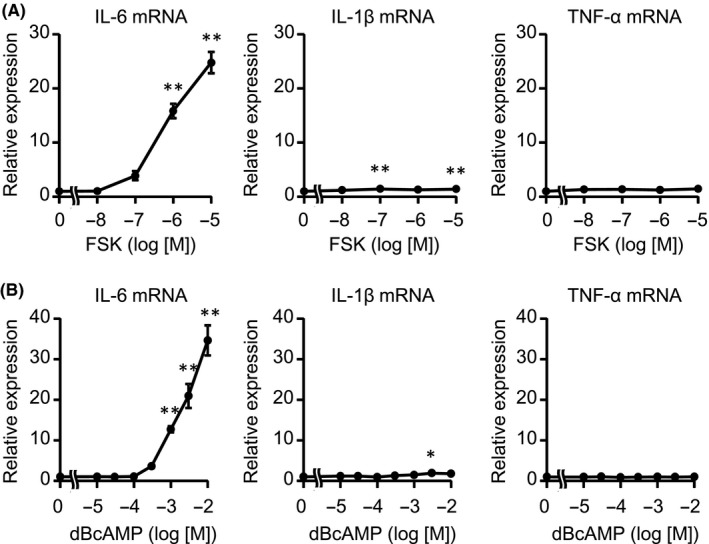
The intracellular increase in cAMP‐induced IL‐6, but not IL‐1β or TNF‐α. A, B) CFs were treated with the indicated concentrations of FSK (A) or dBcAMP (B) for 1 h. The expression of IL‐6, IL‐1β, and TNF‐α mRNA was measured by real‐time RT‐PCR. Results are shown as mean ± SEM (n = 6). *P < .05, **P < .01 vs vehicle by Dunnett test

β2AR transduces signals by activating adenylate cyclase to enhance cAMP level.^19^, ^20^ To examine the effects of cAMP on IL‐6 upregulation in adult murine CFs, we stimulated CFs with forskolin (FSK), an adenylate cyclase activator, or bucladesine (dBcAMP), a cAMP precursor, and measured the mRNA expression of pro‐inflammatory cytokines (Figure 3A,B). As is the case with ISO or SAL, both FSK and dBcAMP dramatically increased the expression of IL‐6 mRNA in a concentration‐dependent manner. These compounds enhanced IL‐1β mRNA to lesser extent, compared with IL‐6, while not TNF‐α mRNA level. Thus, these data demonstrated that the increase in cAMP upregulated IL‐6.

### 3.3 β2‐adrenergic stimulation upregulates IL‐6 through NF‐κB independent pathway

NF‐κB is one of the major transcriptional factors that regulate IL‐6 expression. Interestingly, NF‐κB activation was required to increase IL‐6 expression in neonatal murine CFs.^11^, ^20^ Therefore, we examined whether NF‐κB pathway was activated by ISO. IL‐1β was used as a positive control because the stimulation of IL‐1β activates NF‐κB in neonatal rat CFs ^7^ and we confirmed IL‐6 mRNA increased in adult murine CFs stimulated with IL‐1β as well as ISO (Figure S2).

**Figure 4:**
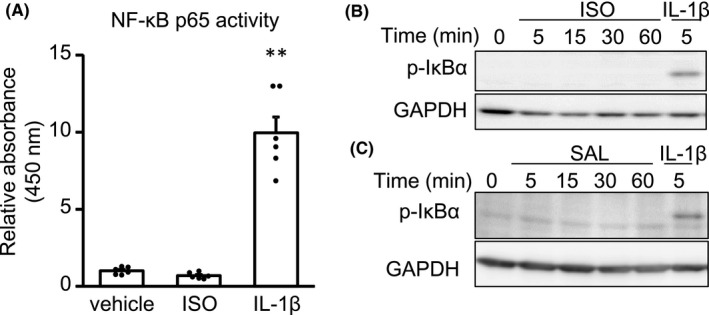
β2‐adrenergic stimulation failed to activate NF‐κB signaling pathway in adult murine CFs. A, CFs were treated with vehicle or ISO (1 μmol/L) for 1 h. IL‐1β (10 ng/mL) was used as a positive control. The transcriptional activity of NF‐κB p65 in CFs was measured by ELISA‐based assay. Results are shown as mean ± SEM (n = 6). **P < .01 vs vehicle by Dunnett test. B, C) CFs were treated with ISO (1 μmol/L) B, SAL (1 μmol/L) C, or IL‐1β (10 ng/mL) for indicated times. The phosphorylation of IκBα in CFs was analyzed by Western blotting with antiphospho‐specific IκBα antibody. The experiments were performed 3 times with similar results. The representative images are shown

NF‐κB is composed of homo‐ or heterodimer, such as p65 and p50. It is well known that p65 subunit of activated NF‐κB translocates into the nucleus and directly promotes gene transcription including *IL‐6*. First, we examined DNA binding activity of NF‐κB p65 in nuclear extracts from CFs, but the activity was not enhanced by ISO (Figure 4A).

IκB binds to NF‐κB and inhibits its activity. In response to extracellular stimuli, IκB is phosphorylated, followed by degradation, leading to the activation of NF‐κB. Therefore, we examined whether IκB is phosphorylated by ISO. Consistent with DNA binding activity assay, Western blotting analysis showed that neither stimulation with ISO nor with SAL enhanced the phosphorylation of IκBα (Figure 4B,C). These data indicate that NF‐κB was not translocated into nuclei and, therefore, failed to regulate transcription. These results were consistent with the finding that the induction of IL‐1β and TNF‐α, well‐known target genes of NF‐κB, was not remarkable in response to ISO, compared with IL‐6.

### 3.4 β2AR signaling upregulates Arid5a to increase IL‐6 mRNA

**Figure 5:**
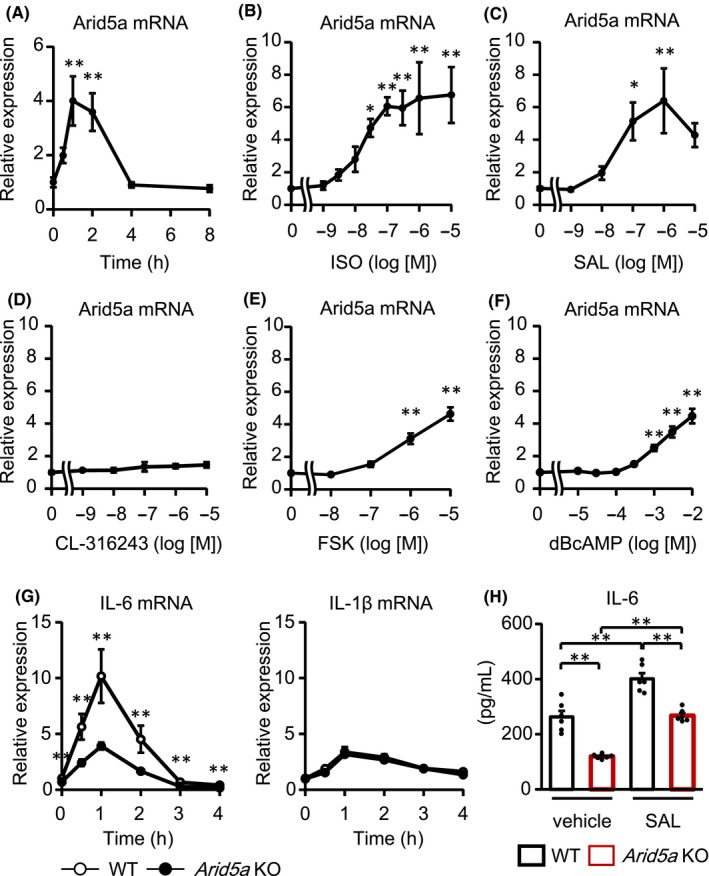
β2AR stimulation upregulated the expression of Aird5a, resulting in IL‐6 mRNA induction. (A) CFs were treated with ISO (10 μmol/L) for indicated times. The expression of Arid5a mRNA was measured by real‐time RT‐PCR. Results are shown as mean ± SEM (n = 6). **P < .01 vs 0 h by Dunnett test. (B‐F) CFs were treated with the indicated concentrations of ISO (B), SAL (C), CL‐316243 (D), FSK (E), or dBcAMP (F) for 1 h. The expression of Arid5a mRNA was measured by real‐time RT‐PCR. Results are shown as mean ± SEM (B: n = 8, C‐F: n = 6). *P < .05, **P < .01 vs vehicle by Dunnett test. G) CFs were isolated from WT mice or *Arid5a* KO mice and treated with or without SAL (1 μmol/L) for indicated times. The expression of IL‐6 and IL‐1β mRNA in CFs was measured by real‐time RT‐PCR. Results are shown as mean ± SEM (n = 6). **P < .01 vs WT by Student's *t* test. H) CFs were isolated from WT mice or *Arid5a* KO mice and treated with or without SAL (1 μmol/L) for 24 h. IL‐6 protein, secreted by CFs in culture medium, was measured by ELISA. Results are shown as mean ± SEM (n = 6). **P < .01 by Tukey‐Kramer test

Thus, we hypothesized that β2AR stimulation stabilizes IL‐6 mRNA and focused on Arid5a, which stabilizes IL‐6 mRNA to increase its expression.^17^ We examined whether the expression of Arid5a mRNA was upregulated by the β2‐adrenergic stimulation in CFs. As is the case with IL‐6 mRNA, Arid5a mRNA was peaked 1 hour after ISO treatment in CFs and increased fourfold compared with baseline (Figure 5A). ISO and SAL increased Arid5a mRNA in a concentration‐dependent manner, but CL‐316243 did not (Figure 5B‐D). ICI‐118551, not CGP20712A or L‐755507, suppressed SAL‐induced Arid5a mRNA upregulation (Figure S3). Moreover, both FSK and dBcAMP also increased Arid5a mRNA (Figure 5E,F), demonstrating that the increase in cAMP resulted in the upregulation of Arid5a mRNA. Furthermore, to address the causality between Arid5a and IL‐6 induction, we isolated CFs from *Arid5a* KO mice and analyzed the mRNA levels of pro‐inflammatory cytokines (Figure 5G). *Arid5a* gene ablation dramatically reduced SAL‐induced upregulation of IL‐6 mRNA, but not that of IL‐1β mRNA. Moreover, the secretion of IL‐6 protein was decreased in *Arid5a* KO CFs compared with WT CFs both in basal and SAL‐treated condition (Figure 5H). The decrease in IL‐6 protein after SAL stimulation was less remarkable than that of mRNA, although the lack of Arid5a led the significant suppression of IL‐6 protein in response to SAL. This discrepancy might result from the difference in time points for analysis. Real‐time PCR was performed 1 hour after SAL stimulation while ELISA was performed 24 hours after the stimulation. Therefore, the amount of IL‐6 protein induced by SAL was partially masked by basal expression. These results indicated that Arid5a plays an important role in IL‐6 expression by β2AR stimulation.

### 3.5 β2‐adrenergic stimulation activates PKA/CREB axis to upregulate Arid5a and IL‐6

**Figure 6:**
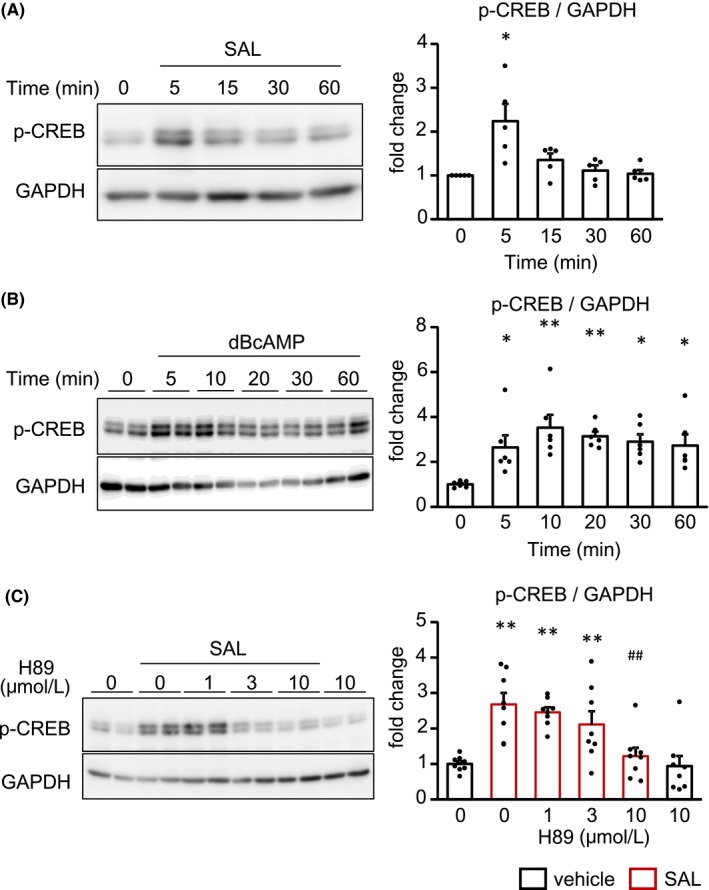
β2‐adrenergic stimulation phosphorylated CREB through cAMP/PKA pathway. A, B, CFs were treated with SAL (1 μmol/L) A, or dBcAMP (3 mmol/L) B, for indicated times. C, CFs were pretreated with the indicated concentrations of H89 for 30 min, followed by the treatment of SAL (1 μmol/L) for 5 min. The phosphorylation of CREB was measured by Western blotting with antiphospho‐CREB antibody. Representative images are shown (left panels). The quantification data are shown as mean ± SEM (A, n = 5, B, n = 6, C, n = 8). *P < .05, **P < .01 vs vehicle, ^##^ *P* < .01 vs DMSO, SAL by Steel test (A) or Dunnett test (B, C) (right panels)

**Figure 7:**
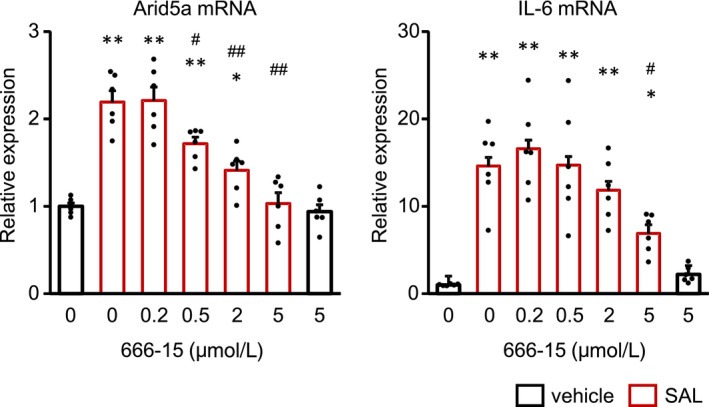
The inhibition of CREB suppressed SAL‐induced mRNA upregulation of Arid5a and IL‐6 expression. CFs were pretreated with the indicated concentrations of 666‐15, an inhibitor of CREB, for 30 min, followed by the treatment with or without SAL (1 μmol/L) for 1 h. The expression of Arid5a and IL‐6 mRNA in CFs was measured by real‐time RT‐PCR. Results are shown as mean ± SEM (n = 6). *P < .05, **P < .01 vs DMSO, vehicle, ^#^ *P* < .05, ^##^ *P* < .01 vs DMSO, SAL by Dunnett test

As cAMP mainly transduces the signal through PKA activation, we examined whether β2AR stimulation phosphorylated CREB Ser133, which is a target of PKA for activation^21^ (Figure 6A‐C). SAL rapidly phosphorylated CREB. Similarly, dBcAMP also phosphorylated CREB 10 minutes after the treatment. Furthermore, SAL‐induced CREB phosphorylation was suppressed with the pretreatment of H89, an inhibitor of PKA, in a concentration‐dependent manner. To exclude the possibility that H89 functions as an antagonist of β2AR,^22^ we examined whether that H89 decreased the phosphorylation of CREB induced by dBcAMP and demonstrated that H89 suppressed CREB phosphorylation (Figure S4). Finally, we investigated whether the CREB phosphorylation was important for the increase in Arid5a and IL‐6 mRNA by using compound 666‐15, a CREB inhibitor. The pretreatment of CFs with 666‐15 blocked SAL‐induced Arid5a and IL‐6 upregulation in a concentration‐dependent manner (Figure 7). From the above results, the β2AR/PKA/CREB axis plays an important role in the expression of Arid5a, resulting in IL‐6 induction as a novel pro‐inflammatory signaling pathway in CFs.

## 4 DISCUSSION

**Figure 8:**
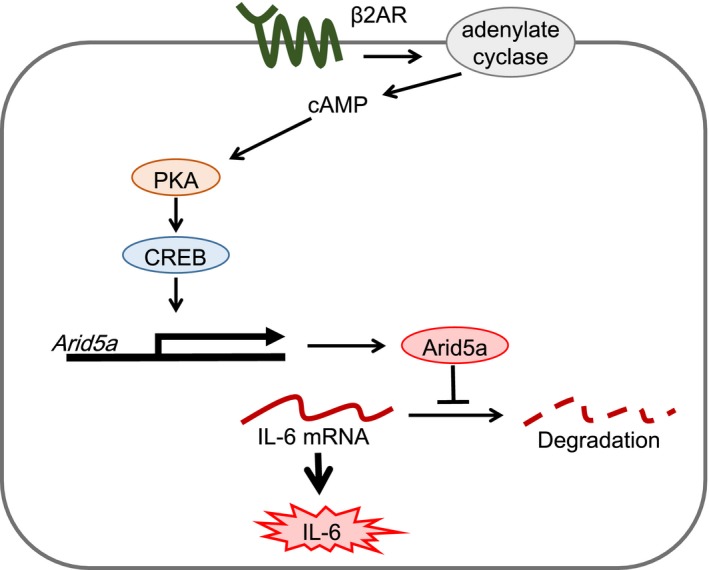
The stimulation of β2AR induces Arid5a through cAMP/PKA/CREB pathway and upregulates IL‐6 expression in adult murine CFs

In this study, we revealed the molecular mechanism of adrenergic stimulation‐medicated induction of IL‐6 in CFs (Figure 8). β2‐adrenergic stimulation increased a pro‐inflammatory cytokine, IL‐6, in adult murine CFs. Both activating adenylate cyclase and cAMP increase also upregulated the expression of IL‐6 mRNA. These stimuli increased Arid5a mRNA as well, and Arid5a was important for the full induction of IL‐6 in response to β2‐adrenergic stimulation. The activation of PKA/CREB pathway resulted in the induction of Arid5a, upregulating IL‐6 expression in NF‐κB independent manner.

Many previous reports have demonstrated that IL‐6/IL‐6R signaling induces cardiac remodeling including myocardial hypertrophy.^2^, ^23^, ^24^ In addition, we have recently reported that β2AR KO mice ameliorated cardiac hypertrophy and dysfunction induced by continuous ISO stimulation and that constitutive activation of PKA in fibroblasts resulted in cardiac hypertrophy.^10^ Taken together with this study, it is possible that β2‐adrenergic stimulation increases IL‐6 release from CFs, contributing to forming cardiac remodeling.

Accumulating evidence has revealed that CFs contribute not only to the maintenance of heart structure but also to cell‐cell communication via paracrine systems. A number of studies have addressed the molecular mechanisms of cytokine production from the viewpoint of transcriptional process and revealed that NF‐κB plays an important role in the expression of pro‐inflammatory cytokines.^25^ Indeed, ISO activates NF‐κB, leading to IL‐6 induction, in neonatal CFs.^12^ Interestingly, in this study, the activation of NF‐κB was not detected in response to ISO stimulation in adult murine CFs either by NF‐κB‐DNA binding assay or by IκB phosphorylation assay. These data indicate that the responses of CFs to extracellular stimuli change with the maturation of the heart and that β2AR signaling pathway is altered during heart growth.

The expression of mRNA is regulated not only by transcription but also by degradation. Since NF‐κB, a major transcriptional factor, is not activated by ISO, we examined whether Arid5a, a stabilizer of IL‐6 mRNA, is involved in IL‐6 upregulation in response to ISO. Importantly, the expression of Arid5a was upregulated by β2AR stimulation and by intracellular increase in cAMP. Moreover, IL‐6 expression was significantly reduced in *Arid5a* KO CFs, indicating that the induction of Arid5a is important for full induction of IL‐6 by β2AR stimulation. Of note, *Arid5a* gene ablation remarkably suppressed IL‐6 expression, but not to the basal level, suggesting that IL‐6 expression is partially regulated in an Arid5a independent manner. Indeed, *IL‐6* gene promoter contains the CRE site, proposing the possibility that CREB directly activated the transcription of IL‐6.^26^, ^27^

Myocardial induction of IL‐6 is closely associated with the progression of heart failure.^3^, ^23^, ^28^ However, heart is constructed of a variety of cells, such as cardiomyocytes, CFs, and endothelial cells; moreover, leucocytes migrate to damaged heart.^29^, ^30^ β‐adrenergic stimulation induces the expression of IL‐6 not only in CFs but also in cardiomyocytes and leucocytes.^24^, ^31^ Importantly, myocardial expression of IL‐6 in response to β‐adrenergic stimulation is complicatedly regulated, depending on cell types. For example, it was previously demonstrated that IL‐6 expression is transcriptionally upregulated in cardiomyocytes through CREB and AP‐1,^32^ while AP‐1 inhibitor failed to suppress IL‐6 upregulation in CFs (data not shown). To address the pathophysiological roles of Arid5a/IL‐6 axis in CFs in cardiac remodeling in vivo, fibroblast‐specific *Arid5a* KO mice should be generated by using the *Periostin‐*promoter driving Cre‐loxP system^33^; however, unfortunately, Arid5a flox mice are unavailable at the present time.

It was reported that Arid5a is induced by NF‐κB^34^; however, ISO failed to activate NF‐κB in adult murine CFs. Thus, we addressed the involvement of CREB, a major transcription factor downstream of cAMP, in Arid5a induction as an alternative pathway. Expectedly, β2AR stimulation enhanced the phosphorylation of CREB in a PKA‐dependent manner. Importantly, compound 666‐15, a CREB inhibitor, inhibited Arid5a and IL‐6 mRNA upregulation, indicating that the transcription of *Arid5a* is regulated by CREB activity as a novel signaling pathway. Although CREB inhibition completely suppressed SAL‐induced Arid5a mRNA upregulation, IL‐6 mRNA still abounded. Therefore, SAL‐induced IL‐6 expression might be regulated partially through the pathway other than CREB/Arid5a axis, although further studies would be needed to identify the pathway.

In conclusion, β2‐adrenergic stimulation regulates Arid5a expression through cAMP/PKA/CREB pathway, contributing to IL‐6 expression. This study proposes that Arid5a regulates IL‐6 expression downstream of adrenergic signal, for the first time. In addition, this is the first demonstration of the importance of Arid5a in cardiac fibroblasts, nonimmune cells. Arid5a could be a therapeutic target for cardiac inflammation in heart failure.

## CONFLICT OF INTEREST

The authors have no conflicts of interest associated with this manuscript.

## Supporting information

Fig S1‐S4

Supplementary Material
