# Supplementary figures and images for: β2‐adrenergic stimulation induces interleukin‐6 by increasing Arid5a, a stabilizer of mRNA, through cAMP/PKA/CREB pathway in cardiac fibroblasts
Source: Pharmacol Res Perspect. 2020 Apr 17;8(2):e00590. doi: 10.1002/prp2.590 (PMC7164407; doi:10.1002/prp2.590)

Supplementary Figure 1

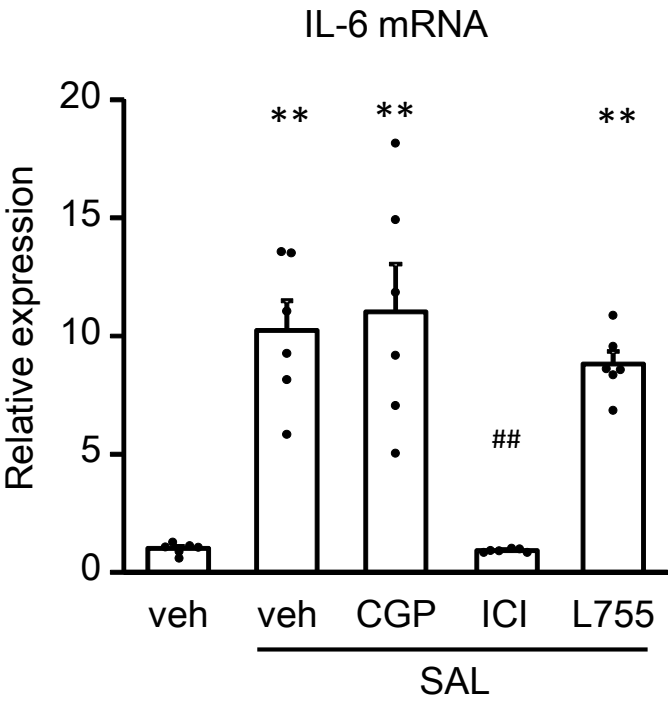

Supplementary Figure 2

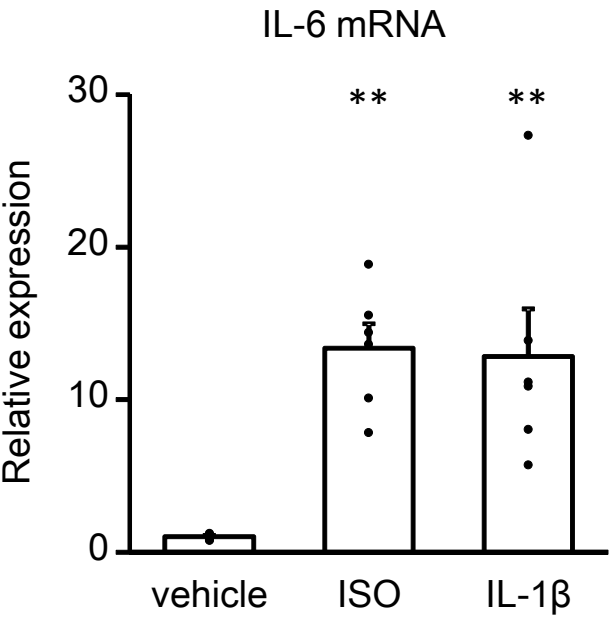

Supplementary Figure 3

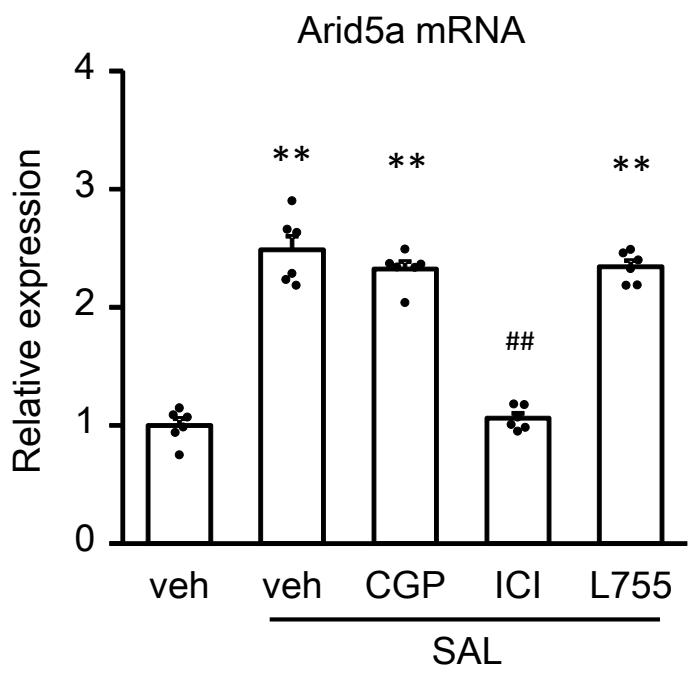

Supplementary Figure 4

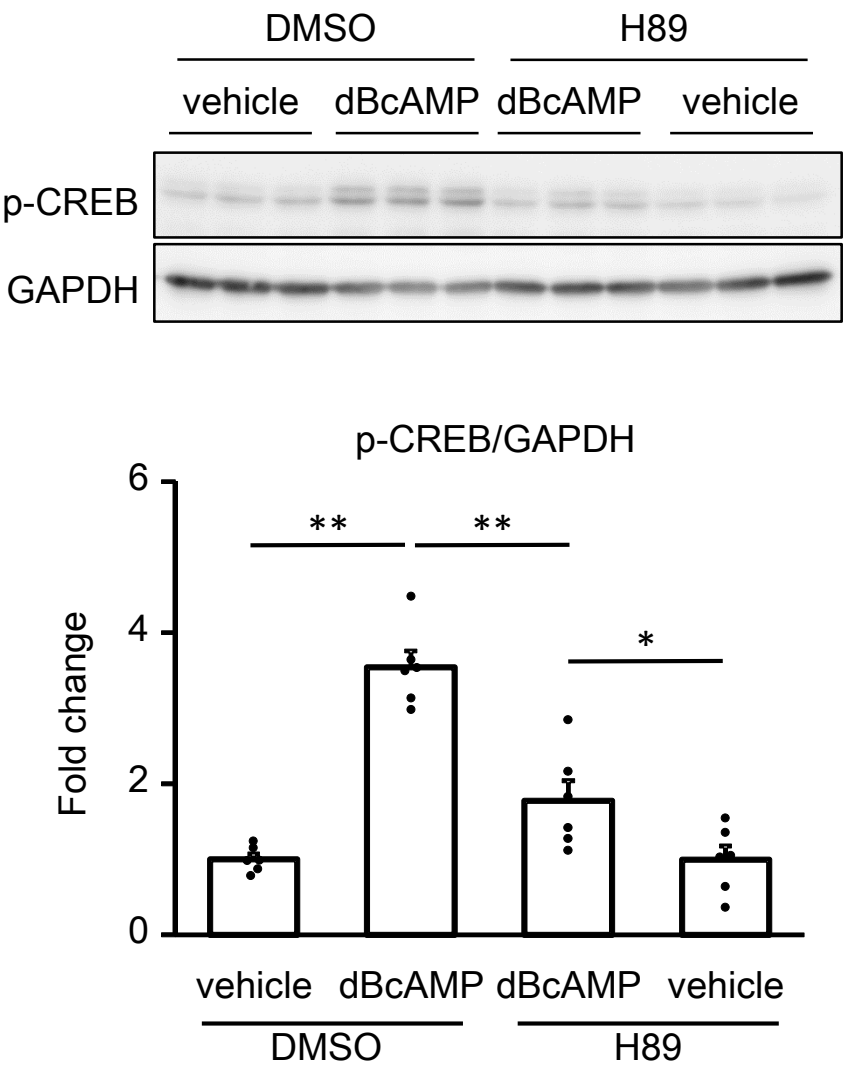

Supplement: Supplementary file 1 — Fig S1‐S4 [file PRP2-8-e00590-s001.pdf]
