# Supplementary material for: β2‐adrenergic stimulation induces interleukin‐6 by increasing Arid5a, a stabilizer of mRNA, through cAMP/PKA/CREB pathway in cardiac fibroblasts
Source: Pharmacol Res Perspect. 2020 Apr 17;8(2):e00590. doi: 10.1002/prp2.590 (PMC7164407; doi:10.1002/prp2.590)
Supplement: Supplementary file 2 — Supplementary Material [file PRP2-8-e00590-s002.docx]

**β2-adrenergic stimulation induces interleukin-6 by increasing Arid5a, a stabilizer of mRNA, through cAMP/PKA/CREB pathway in cardiac fibroblasts**

**Supporting Information**

Supplementary Figure 1

SAL-induced IL-6 mRNA upregulation suppressed by the inhibitor of β2AR, not β1AR or β3AR.

CFs were pretreated with the indicated concentrations of CGP20712A (CGP, 1 μM), a selective β1AR inhibitor, ICI-118551 (ICI, 1 μM), a selective β2AR inhibitor, or L-755507 (L755, 1 μM), a selective β3AR inhibitor, for 30 minutes, followed by the treatment with or without SAL (1 μM) for 1 hour. The expression of IL-6 mRNA in CFs was measured by real-time RT-PCR. Results are shown as mean ± SEM (n = 6). ***P* < 0.01 vs vehicle, ^##^*P* < 0.01 vs vehicle, SAL by Tukey-Kramer test.

Supplementary Figure 2

The stimulation with IL-1β also increased IL-6 mRNA as much as ISO stimuli.

CFs were treated with ISO (1 μM) or IL-1β (10 ng/mL) for 1 hour. The expression of IL-6 mRNA was measured by real-time RT-PCR. Results are shown as mean ± SEM (n = 6). ***P* < 0.01 vs vehicle by Dunnett test.

Supplementary Figure 3

SAL-induced Arid5a mRNA upregulation suppressed by the inhibitor of β2AR, not β1AR or β3AR.

CFs were pretreated with the indicated concentrations of CGP (1 μM), ICI (1 μM), or L755 (1 μM) for 30 minutes, followed by the treatment with or without SAL (1 μM) for 1 hour. The expression of Arid5a mRNA in CFs was measured by real-time RT-PCR. Results are shown as mean ± SEM (n = 6). ***P* < 0.01 vs vehicle, ^##^*P* < 0.01 vs vehicle, SAL by Tukey-Kramer test.

Supplementary Figure 4

H89 suppressed the phosphorylation of CREB induced by dBcAMP.

CFs were pretreated with or without H89 (10 μM) for 30 minutes, followed by the treatment of dBcAMP (3 mM) for 10 minutes. The phosphorylation of CREB was measured by western blotting with anti-phospho-CREB antibody. Representative images are shown (upper panels). The quantification data are shown as mean ± SEM (n = 6). **P* < 0.05, ***P* < 0.01 by Tukey-Kramer test (lower panels).
